# Social inequalities in eligibility rates and use of the Australian National Disability Insurance Scheme, 2016–22: an administrative data analysis

**DOI:** 10.5694/mja2.52594

**Published:** 2025-02-16

**Authors:** George Disney, Yi Yang, Peter Summers, Alexandra Devine, Helen Dickinson, Anne M Kavanagh

**Affiliations:** ^1^ Melbourne Disability Institute the University of Melbourne Melbourne VIC; ^2^ Centre for Health Equity the University of Melbourne Melbourne VIC; ^3^ Centre for Health Policy the University of Melbourne Melbourne VIC; ^4^ The University of New South Wales Canberra ACT

**Keywords:** Disabled persons, Socioeconomic factors, Gender identity

## Abstract

**Objectives:**

To assess differences in eligibility rates and use of the National Disability Insurance Scheme (NDIS).

**Study design:**

Analysis of NDIS unit‐record administrative data.

**Setting, participants:**

Applicants for NDIS support aged 7 years or older, 1 July 2016 – 31 August 2022 (eligibility analysis); active NDIS participants aged 7 years or older on 31 August 2022 (plan size and spending analyses).

**Main outcome measures:**

Differences in NDIS eligibility rates by broad age group (under 55 years *v* 55 years or older), gender (girls and women *v* other applicants), and residential socio‐economic status (three lowest deciles of the Index of Relative Socioeconomic Disadvantage *v* other areas); differences in NDIS personal plan size (allocation) and spending (use) by gender and residential socio‐economic status.

**Results:**

During 2016–22, 705 594 people aged 7 years or older had applied for NDIS support; 485 676 applicants with recorded decisions were included in our analysis (393 152 eligible, 92 524 ineligible). Eligibility rates were highest for applicants with brain injury or stroke, intellectual disability, or autism (900 or more per 1000 applicants), and only minor inequalities by socio‐demographic group were evident. Eligibility rates were lower for applicants with physical disability, psychosocial disability, or unclassified (other) disability (60–75%). Eligibility inequalities were most marked for people with physical disability, with fewer approvals for women and girls than men and boys (145 [95% confidence interval {CI}, 138 − 152] fewer approvals per 1000 applicants), for people aged 55 years or older than for younger applicants (235 [95% CI, 227–242] fewer approvals per 1000 applicants), and for people from lower socio‐economic status areas than for those from other areas (86 [95% CI, 78–93] fewer approvals per 1000 applicants). The eligibility rate for applicants with psychosocial disability was lower for women and girls than men and boys (83 [95% CI, 77–89] fewer approvals per 1000 applicants). Inequalities in plan sizes and spending by socio‐economic group and gender for the 312 268 active participants at 31 August 2022 were smaller.

**Conclusions:**

Women and girls and applicants over 55 years of age or living in socio‐economically disadvantaged areas with certain disability types are less likely to be deemed eligible for NDIS support than other applicants. Inequalities in plan allocation and use of personal NDIS budgets are less marked. Changes to NDIS eligibility processes could reduce these inequalities.

**The known**: Qualitative studies have identified inequalities in access to the National Disability Insurance Scheme (NDIS) and the purchase of appropriate disability services. However, the inequalities have not been quantified.

**The new**: We found inequalities in NDIS access for applicants with physical, psychosocial, and unclassified disability types, particularly for women and girls. Among people deemed eligible for support, social inequalities in the allocation and use of NDIS personal budgets are less marked.

**The implications**: Social inequalities in the operation of the NDIS, particularly in assessing eligibility for the scheme, should be further investigated to determine whether specific disability and social groups are systematically disadvantaged.

The introduction of the National Disability Insurance Scheme (NDIS) was one of the largest social policy reforms ever undertaken in Australia. NDIS expenditure during 2023–24 is projected to total $41.9 billion, providing individualised support budgets for more than 600 000 participants with permanent and significant disability or who meet early intervention criteria.^1^, ^2^

The NDIS replaced a largely block‐funded disability care system that was criticised as underfunded, unfair, and fragmented, providing only limited choice and limited access to appropriate support for people with disability.^3^ The self‐directed nature of NDIS support aims to remedy these problems by providing participants with individual budgets to purchase the services they need. However, concerns have grown that social inequalities could influence access to the scheme and limit choices and control for NDIS participants. Further, higher funding for the NDIS than for aged care could lead to inequitable access to social services for people approaching the NDIS cut‐off age of 65 years.^4^

Intersections of disability with broader social inequalities, such as poverty, could also hinder full participation in the NDIS.^5^ People with disability do not have the same access to public services and health care as other people, in part because they cannot afford the out‐of‐pocket costs.^6^ As access to the NDIS is partly based on medical evidence provided by treating health professionals, the cost of proving eligibility may be too high for people with limited financial resources.^7^ Further, women may be disadvantaged by a disability support system overly influenced by a medical system that has historically favoured men.^8^

Published quantitative research into inequalities in access to and use of the NDIS is limited. Analyses of aggregated data^9^ can be subject to bias and socio‐demographic confounders. As the NDIS is a national scheme with a single coherent data infrastructure, it provides a unique opportunity for quantifying inequalities in the operation of a self‐directed disability support system. We therefore used unit record NDIS data to investigate whether eligibility rates for the NDIS differ by gender, residential socio‐economic status, or broad age group; and, for people deemed eligible, whether the allocation and use of NDIS support differs by gender and residential socio‐economic status.

## Methods

For our retrospective analysis of administrative data, we obtained a tailored data release for the period 1 July 2016 – 31 August 2022 from the National Disability Insurance Agency. Records for each applicant and participant include socio‐demographic and disability information, and information about allocated funding and payments to service providers (NDIS support types: Supporting Information, table 1).

### Study sample

We included data for applicants and participants aged 7 years or older for whom NDIS eligibility decisions were recorded. We excluded applicants under 7 years of age because the eligibility criteria are different for this age group.

In the NDIS eligibility analysis, we included applicants with an eligibility decision based on their most recent access request. We excluded cancelled, withdrawn, in‐progress, and ceased applications (Box 1). In the analysis of allocation and use of NDIS plans, we included each active participant's most recent completed plan for more than 180 days’ support to reflect their current support needs. We reported the allocation (plan size) and use (spending) for all types of support and other services as annualised dollar amounts.

Box 1 **National Disability Insurance Scheme (NDIS) access request decision types**

**Table:** 

Decision type	Typical reasons for decision
Access met	Access request was successful. Application was assessed and deemed eligible.
Access not met	Access request was unsuccessful. Application was assessed and deemed ineligible.
Withdrawn	Application withdrawn by the applicant.
Cancelled	Application cancelled and not assessed, because: the applicant did not return an access request form; the applicant did not provide required evidence for the access request; or National Disability Insurance Agency was unable to contact the applicant.
In progress	Application is in progress.
Access revoked or ceased	The applicant no longer has a need for NDIS support.

### Socio‐demographic groups of interest

We investigated differences in eligibility for and the use of NDIS funding in three groups:

- women and girls (self‐reported gender);
- people living in socio‐economically disadvantaged areas (three lowest deciles of the Index of Relative Socioeconomic Disadvantage [IRSD]);^10^
- people aged 55 years or older at the eligibility decision date. All applicants under 65 years of age should have equal access to the NDIS, but as support needs increase with age, we examined only age‐related inequality in NDIS eligibility, not in the allocation and use of plans.

### Primary disability groups

We estimated inequalities by NDIS primary disability group, mapped to the broader disability groups used by the Australian Bureau of Statistics in the 2018 Survey of Disability, Ageing and Carers^11^, ^12^ (Box 2).

Box 2 **Comparison of primary disability types in the National Disability Insurance Scheme (NDIS) and in the Survey of Disability, Ageing and Carers**

**Table:** 

Definition	National Disability Insurance Scheme^13^	Survey of Disability, Ageing and Carers^11^
Definition of disability	Permanent or significant loss or reduction in functional capacity to undertake one or more of six core activities: communication, social interaction, learning, mobility, self‐care, or self‐management. Loss or reduction in functioning is attributed to one or more intellectual, cognitive, neurological, sensory, or physical impairments, or to one or more impairments attributable to a psychiatric condition.	At least one limitation, restriction, or impairment of the following ten everyday activities for at least six months: self‐care, mobility, communication, cognitive or emotional tasks, health care, reading or writing tasks, transport, household chores, property maintenance, and meal preparation. Survey of Disability, Ageing and Carers also assess restrictions in two other life areas: schooling and employment.
Severity of disability	Severity assessed with functional assessment tools (eg, DSM‐5, Pedi‐CAT, WHODAS, CANS). The scores from different tools are standardised (scale, 1–15), with lower values indicating lesser severity (higher functional capacity).	Severe or profound disability is defined as sometimes or always needing help with one or more of three core activities (self‐care, mobility, communication)
Disability groups	NDIS primary disability types grouped to match broader disability groups used by Survey of Disability, Ageing and Carers: sensory disability: visual impairment, hearing impairment, other sensory or speech impairment; autism;* intellectual disability other than autism: intellectual disability, trisomy 21, developmental delay, global developmental delay; physical disability: cerebral palsy, spinal cord injury, other physical disability; psychosocial disability; head injury or stroke: acquired brain injury, stroke; other disability: other, multiple sclerosis, other neurological disability.	Survey of Disability, Ageing and Carers does not determine a single primary disability group; people are included in two or more of the following disability groups: sensory (sight, hearing speech); intellectual (difficulty learning or understanding); physical (including breathing difficulties, chronic or recurrent pain, incomplete use of limbs); psychosocial (including nervous or emotional conditions, mental illness, memory problems, and social or behavioural difficulties); head injury, stroke or acquired brain injury; other restrictions in everyday activities due to other long‐term conditions or ailments.

CANS = Care and need scale; DSM‐5 = the Diagnostic and Statistical Manual of Mental Disorders, fifth edition; Pedi‐CAT = Pediatric Evaluation of Disability Inventory‐Computer Adaptive Test; WHODAS = World Health Organization Disability Assessment Schedule.

* Examined separately from other intellectual disabilities because people with autism comprise the largest primary disability group in the NDIS.

### Statistical analysis

Eligibility inequality was defined as a difference between two groups in the number of eligible applications per 1000 access requests. Inequalities in allocation and use of support services were defined as differences in mean plan size and spending (in dollars).

We used a three‐step approach to assess differences in NDIS eligibility (Box 3) and plan size and spending (Box 4). First, we constructed a causal diagram depicting the assumed relationship between a defined group of people and the outcomes of interest.^14^, ^15^ Causal diagrams help decisions about which variables influence the effect of interest and should therefore be considered in analyses. Second, we specified target trials that assess the effects indicated by the pathways in the causal diagram.^16^ Third, we used NDIS data to emulate the target trials,^16^ using Monte Carlo simulation‐based *g*‐computation to estimate the magnitude of inequalities.^17^

Box 3 **Estimation of inequalities in access to National Disability Insurance Scheme (NDIS)**

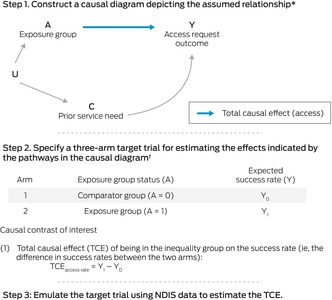

* Based on evidence from published qualitative research and expert opinion. C = confounders that influence or co‐exist with the characteristic of the inequality group (A) that affect the outcome Y; U = unmeasured factors that influence the inequality group (A) and the confounders (C).

† The trial comprises two arms: the comparator arm and the exposure arm. Further details: Supporting Information, supplementary methods, section 2.

Box 4 **Estimation of inequalities in the allocation and use of National Disability Insurance Scheme (NDIS) services and support**

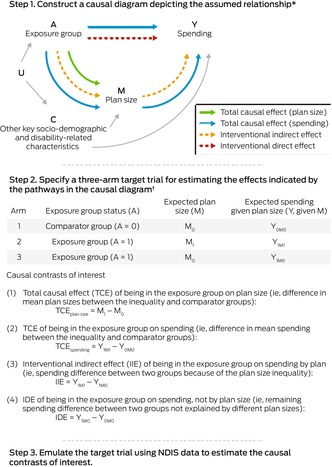

* Based on evidence from published qualitative research and expert opinion. C = confounders that influence or co‐exist with the characteristic of the inequality group (A) that affect the outcome Y; U = unmeasured factors that influence the inequality group (A) and the confounders (C).

† The trial comprises three arms: the comparator arm, the exposure arm, and the exposure arm with an intervention that shifts its plan size distribution to match that of the comparator arm. Further details: Supporting Information, supplementary methods, section 2.

For the first research question, the total causal effect, adjusted for previous source of disability support, is an estimate of eligibility inequality. For the second research question, the total causal effects are estimates of plan size and spending inequalities, adjusted for age, remoteness category (major cities or regional/rural/remote),^18^disability severity, years of participation in the NDIS, participation in disability accommodation programs, and previous source of disability support. For the second research question, we assumed that allocated funds are a mediator of the relationship between the group of interest and spending (Box 4). That is, differences in spending could be attributable to differences in the allocated funding for people in the two groups. We therefore decomposed the total causal effect for spending into interventional direct and indirect effects.^19^ The interventional indirect effects reflect how spending by people in the group of interest would change were the mean plan size the same as that of the comparator group; it quantifies the extent to which spending differences are attributable to differences in budget allocation (further details: Supporting Information, table 2).

Analyses were performed in R using code adapted from Moreno‐Betancur and colleagues.^20^ Our code is available at https://github.com/YiYang368/NDIS_Inequalities.

### Ethics approval

The University of Melbourne human research ethics committee approved the study (2023‐13261‐39232‐3).

## Results

### Eligibility for NDIS support

During 1 July 2016 – 31 August 2022, 705 594 people aged 7 years or older had applied for NDIS support; after excluding applications that were incomplete, cancelled, or withdrawn (136 199, 19%), in progress (52 584, 7%), or revoked or ceased (30 444, 4%), and a further 691 applicants for whom socio‐demographic information was incomplete (0.1%), we included 485 676 applicants with recorded decisions in our analysis: 393 152 applicants deemed eligible for the NDIS and 92 524 deemed ineligible (Supporting Information, figure 1).

Of the 485 676 applicants, 126 268 (26%) were aged 7–14 years and 100 387 (21%) 55–64 years, 210 274 (43%) were women or girls, and 150 472 (31%) lived in socio‐economically disadvantaged areas; 286 376 applicants (59%) had not previously received government disability support. The most frequent primary disabilities were autism (125 916, 26%), intellectual disability other than autism (91 542, 19%), and psychosocial disability (82 525, 17%) (Box 5).

Box 5 **National Disability Insurance Scheme (NDIS) applicants included in the eligibility analysis: socio‐demographic and disability group characteristics**

**Table:** 

Characteristics	Number
All applicants	485 676
**Socio‐demographic characteristics**	
Age group, access request decision (years)	
7–14	126 268 (26.0%)
15–18	41 647 (8.6%)
19–24	36 463 (7.5%)
25–34	50 967 (10.5%)
35–44	56 232 (11.6%)
45–54	73 712 (15.2%)
55–64	100 387 (20.7%)
Gender (women and girls)	210 274 (43.3%)
Indigenous Australians	32 866 (6.8%)
Living in socio‐economically disadvantaged areas*	150 472 (31.0%)
Living in regional or remote areas	159 416 (32.8%)
**Disability group**	
Sensory	36 197 (7.5%)
Hearing impairment	22 042 (4.5%)
Visual impairment	10 433 (2.1%)
Other sensory/speech	3722 (0.8%)
Autism	125 916 (25.9%)
Intellectual, other than autism	91 542 (18.8%)
Developmental delay	337 (0.1%)
Global developmental delay	155 (< 0.1%)
Trisomy 21	9369 (1.9%)
Intellectual disability	81 681 (16.8%)
Physical	66 449 (13.7%)
Cerebral palsy	13 751 (2.8%)
Spinal cord injury	6013 (1.2%)
Other physical	46 685 (9.6%)
Psychosocial	82 525 (17.0%)
Brain injury or stroke	28 164 (5.8%)
Acquired brain injury	18 248 (3.8%)
Stroke	9916 (2.0%)
Other	54 883 (11.3%)
Multiple sclerosis	10 929 (2.3%)
Other neurological	26 377 (5.4%)
Other	17 577 (3.6%)
**Previous source of support**	
Australian government	37 478 (7.7%)
State government	161 822 (33.3%)
No prior support	286 376 (59.0%)

* Three lowest deciles of the Index of Relative Socioeconomic Disadvantage (IRSD).^10^

Eligibility rates were highest for applicants with brain injury or stroke, intellectual disability, or autism (900 or more approvals per 1000 applicants). Access inequalities were smallest for people in these groups, except that applicants with autism aged 55 years or older were less likely to be deemed eligible than younger participants with autism (90 fewer approvals per 1000 applicants; 95% confidence interval [CI], 67–114 fewer approvals per 1000 applicants) (Box 6).

Box 6 Assessment of inequalities in eligibility for the National Disability Insurance Scheme (NDIS), by disability group*

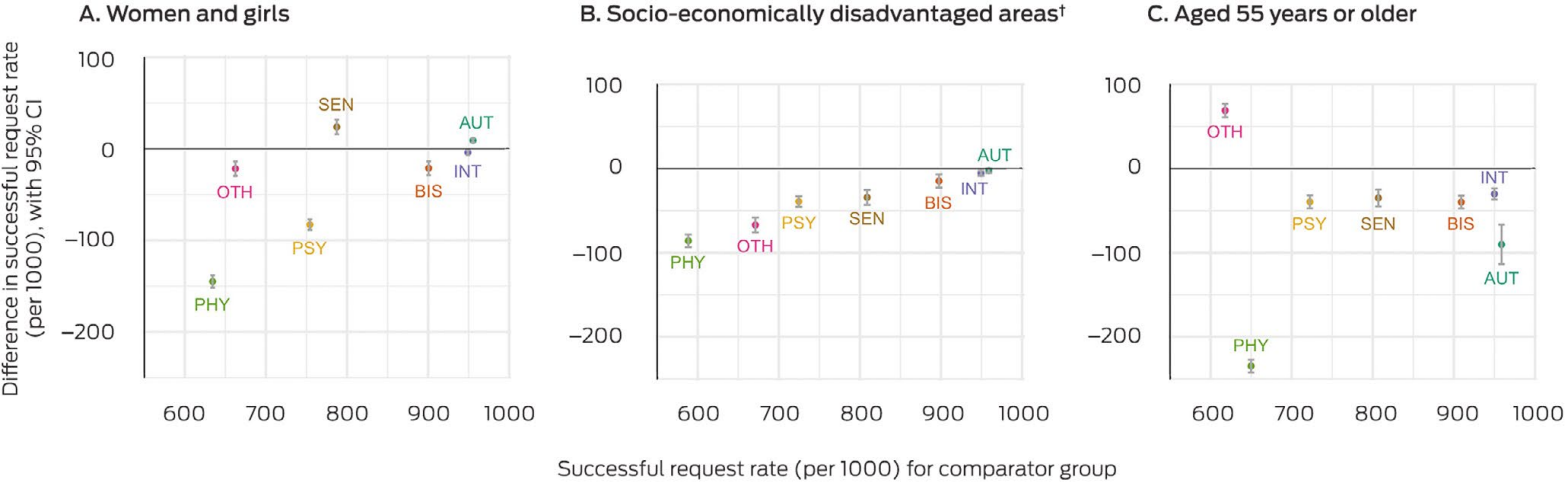

AUT = autism; BIS = brain injury or stroke; CI = confidence interval; INT = intellectual disability; OTH = other disabilities; PHY = physical disability; PSY = psychosocial disability; SEN = sensory disability.

* Total causal effect, adjusted for prior source of disability support (Australian government, state government, no prior support). The number of eligible access requests per 1000 applications for the comparator group is plotted against the x‐axis, the difference in eligibility rates between the exposure and comparator groups against the y‐axis. The data underlying these graphs are reported in the Supporting Information, table 3.

† Three lowest deciles of the Index of Relative Socioeconomic Disadvantage (IRSD).^10^

Eligibility rates were lower for applicants with physical disability, psychosocial disability, or unclassified (other) disability (590–750 approvals per 1000 applicants). Eligibility inequalities were most marked for people with physical disability, with fewer approvals for women and girls than men and boys (145 [95% CI, 138−155] fewer approvals per 1000 applicants) and for people aged 55 years or older than for younger applicants (235 [95% CI, 227–242] fewer approvals per 1000 applicants). The eligibility rate for women and girls with psychosocial disability was lower than for men and boys (83 [95% CI, 77–89] fewer approvals per 1000 applicants). Eligibility rates for applicants in socio‐economically disadvantaged areas were generally lower than for other applicants, particularly for those with physical, psychosocial, or other disability (Box 6).

### Social inequalities in the allocation and use of NDIS support

Among the 312 268 active NDIS participants on 31 August 2022 (completed plan longer than 180 days), the most frequent primary disabilities were autism (88 609 people, 28%), intellectual disability (76 248, 24%), and psychosocial disability (45 056, 14%) (Supporting Information, table 4). The median age group for female participants was 35–44 years, and for other active participants 25–34 years; the proportion of women and girls with autism (18.7%) was smaller than for other active participants (35.0%). The age distribution of active participants living in lower socio‐economic status was similar to that for those in other areas, as were the proportions by disability type; the proportion of people in lower socio‐economic status areas living in regional or remote areas (50.0%) was larger than for other participants (24.9%) (Box 7).

Box 7 **National Disability Insurance Scheme (NDIS) participants included in the plan size and spending analysis: socio‐demographic and disability characteristics**

**Table:** 

Characteristics	Women and girls	Others	Living in socio‐economically disadvantaged areas*	Other areas
All applicants	127 125	185 143	93 341	218 927
**Socio‐demographic characteristics**				
Age group, plan start (years)				
7–14	20 258 (15.9%)	45 478 (24.6%)	18 298 (19.6%)	47 438 (21.7%)
15–18	10 174 (8.0%)	20 333 (11.0%)	8978 (9.6%)	21 529 (9.8%)
19–24	11 983 (9.4%)	21 291 (11.5%)	10 200 (10.9%)	23 074 (10.5%)
25–34	15 594 (12.3%)	22 011 (11.9%)	11 425 (12.2%)	26 180 (12.0%)
35–44	16 648 (13.1%)	19 759 (10.7%)	11 098 (11.9%)	25 309 (11.6%)
45–54	21 445 (16.9%)	23 344 (12.6%)	13 771 (14.8%)	31 018 (14.2%)
55–64	31 023 (24.4%)	32 927 (17.8%)	19 571 (21.0%)	44 379 (20.3%)
Gender (women and girls)	—	—	37 663 (40.3%)	89 462 (40.9%)
Indigenous Australians	7616 (6.0%)	12 247 (6.6%)	8810 (9.4%)	11 053 (5.0%)
Living in socio‐economically disadvantaged areas*	37 663 (29.6%)	55 678 (30.1%)	—	—
Living in regional or remote areas	41 124 (32.3%)	60 162 (32.5%)	46 692 (50.0%)	54 594 (24.9%)
**Disability group**				
Sensory	10 499 (8.3%)	10061 (5.4%)	5887 (6.3%)	14673 (6.7%)
Hearing impairment	6459 (5.1%)	5648 (3.1%)	3412 (3.7%)	8695 (4.0%)
Visual impairment	3811 (3.0%)	3876 (2.1%)	2259 (2.4%)	5428 (2.5%)
Other sensory/speech	229 (0.2%)	537 (0.3%)	216 (0.2%)	550 (0.3%)
Autism	23 800 (18.7%)	64 809 (35.0%)	24 158 (25.9%)	64 451 (29.4%)
Intellectual, other than autism	33 125 (26.1%)	43 123 (23.3%)	25 534 (27.4%)	50 714 (23.2%)
Developmental delay*	27 (< 0.1%)	48 (< 0.1%)	25 (< 0.1%)	50 (< 0.1%)
Global developmental delay*	< 15	32 (< 0.1%)	16 (< 0.1%)	25 (< 0.1%)
Trisomy 21	4099 (3.2%)	4978 (2.7%)	2443 (2.6%)	6634 (3.0%)
Intellectual disability	28 990 (22.8%)	38 065 (20.6%)	23 050 (24.7%)	44 005 (20.1%)
Physical	14 751 (11.6%)	18184 (9.8%)	9898 (10.6%)	23 037 (10.5%)
Cerebral palsy	5553 (4.4%)	6816 (3.7%)	3471 (3.7%)	8898 (4.1%)
Spinal cord injury	1409 (1.1%)	3529 (1.9%)	1393 (1.5%)	3545 (1.6%)
Other physical	7789 (6.1%)	7839 (4.2%)	5034 (5.4%)	10 594 (4.8%)
Psychosocial	21 977 (17.3%)	23 079 (12.5%)	13 465 (14.4%)	31 591 (14.4%)
Brain injury or stroke	7563 (5.9%)	12 913 (7.0%)	6631 (7.1%)	13845 (6.3%)
Acquired brain injury	4674 (3.7%)	9195 (5.0%)	4413 (4.7%)	9456 (4.3%)
Stroke	2889 (2.3%)	3718 (2.0%)	2218 (2.4%)	4389 (2.0%)
Other	15 410 (12.1%)	12 974 (7.0%)	7768 (8.3%)	20 616 (9.4%)
Multiple sclerosis	6248 (4.9%)	2153 (1.2%)	2037 (2.2%)	6364 (2.9%)
Other neurological	7645 (6.0%)	8935 (4.8%)	4629 (5.0%)	11 951 (5.5%)
Other	1517 (1.2%)	1886 (1.0%)	1102 (1.2%)	2301 (1.1%)
**Previous source of support**				
Australian government	11 175 (8.8%)	17307 (9.3%)	8898 (9.5%)	19 584 (8.9%)
State government	58 441 (46.0%)	85 004 (45.9%)	43 448 (46.5%)	99 997 (45.7%)
No prior support	57 509 (45.2%)	82 832 (44.7%)	40 995 (43.9%)	99 346 (45.4%)
**Disability severity score**				
1–5	19 209 (15.1%)	27 438 (14.8%)	13 277 (14.2%)	33 370 (15.2%)
6–10	61 643 (48.5%)	98 393 (53.1%)	48 710 (52.2%)	111 326 (50.9%)
11–15	46 273 (36.4%)	59 312 (32.0%)	31 354 (33.6%)	74 231 (33.9%)
**Disability care history**				
Time in NDIS prior to current plan (years)				
One or less	43 333 (34.1%)	61 004 (32.9%)	30 756 (33.0%)	73 581 (33.6%)
More than 1 to 2	29 572 (23.3%)	43 318 (23.4%)	21 210 (22.7%)	51 680 (23.6%)
More than 2 to 3	26 240 (20.6%)	38 589 (20.8%)	19 875 (21.3%)	44 954 (20.5%)
More than 3 to 4	19 090 (15.0%)	29 163 (15.8%)	15 170 (16.3%)	33 083 (15.1%)
More than 4	8890 (7.0%)	13 069 (7.1%)	6330 (6.8%)	15 629 (7.1%)
Disability accommodation programs				
Ever been a Younger People in Residential Aged Care Strategy participant	2420 (1.9%)	2911 (1.6%)	1583 (1.7%)	3748 (1.7%)
Ever received funds for supported independent living	12 444 (9.8%)	18 649 (10.1%)	8898 (9.5%)	22 195 (10.1%)
Ever received funds for specialist disability accommodation	9740 (7.7%)	14 097 (7.6%)	6715 (7.2%)	17 122 (7.8%)
Received a trial plan prior to 30 June 2016	6712 (5.3%)	10 770 (5.8%)	4163 (4.5%)	13 319 (6.1%)

* Delay in a child's development that results in functional limitation to undertake everyday activities. Children with developmental delay in the NDIS early intervention program are generally re‐assessed for full scheme eligibility because of any other disability after they turn six years old. Consequently, the numbers of participants aged 7 years or older with developmental delay or global developmental delay recorded as the primary disability were relatively small. Cell counts lower than 15 are suppressed for privacy reasons.

Overall, plan allocations were largest for participants with brain injury and stroke, physical disability, other disabilities, or intellectual disability groups ($119 000–154 000). Differences in mean plan size by gender or socio‐economic disadvantage group were small (less than $9000) (Box 8).

Box 8 Assessment of inequalities in National Disability Insurance Scheme (NDIS plan size), by disability group*

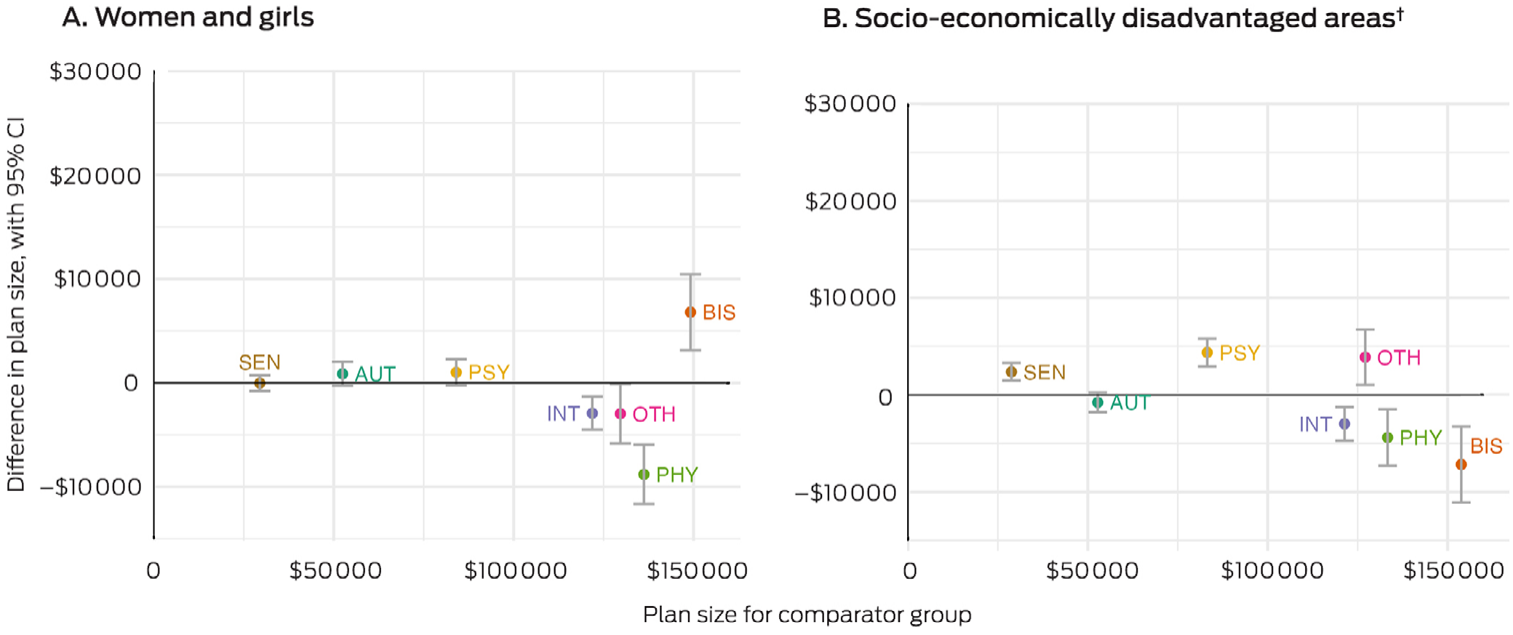

AUT = autism; BIS = brain injury or stroke; CI = confidence interval; INT = intellectual disability; OTH = other disabilities; PHY = physical disability; PSY = psychosocial disability; SEN = sensory disability.

* Total causal effect, adjusted for age, residential remoteness, disability severity, time in the NDIS, participation in disability accommodation programs or received NDIS trial plans, and source of disability support before the NDIS (Australian government, state government, no prior support), as well as for other exposures of interest. Plan sizes for the comparator group are plotted against the x‐axis, the difference in plan sizes between the exposure and comparator groups against the y‐axis. The data underlying these graphs are reported in the Supporting Information, table 5.

† Three lowest deciles of the Index of Relative Socioeconomic Disadvantage (IRSD).^10^

Differences in mean spending by gender or socio‐economic disadvantage group generally reflected those for plan allocation. However, for women and girls, the direct effect was greater than the indirect effect for most disability groups, most markedly for people with psychosocial disability; that is, NDIS spending by women and girls with psychosocial disability was marginally higher than expected, given their plan sizes (Box 9).

Box 9 Assessment of inequalities in National Disability Insurance Scheme (NDIS) spending, by disability group*

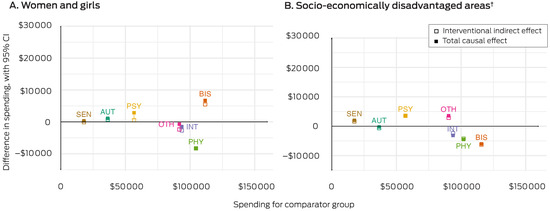

AUT = autism; BIS = brain injury or stroke; INT = intellectual disability; OTH = other disabilities; PHY = physical disability; PSY = psychosocial disability; SEN = sensory disability.

* Adjusted for age, residential remoteness, disability severity, time in the NDIS, participation in disability accommodation programs or received NDIS trial plans, and source of disability support before the NDIS (Australian government, state government, no prior support), as well as for other exposures of interest. Spending for the comparator group are plotted against the x‐axis, the difference in spending between the exposure and comparator groups against the y‐axis. The solid squares denote overall differences in spending (total causal effect), the clear squares differences in spending attributable to differences in plan size (intervention indirect effect); interventional direct effects are the differences between the total causal effects and the interventional indirect effects. The 95% confidence intervals are omitted to enhance clarity. The data underlying these graphs are reported in the Supporting Information, table 5.

† Three lowest deciles of the Index of Relative Socioeconomic Disadvantage (IRSD).^10^

## Discussion

We found that NDIS eligibility rates were lower in certain disability groups for people over 55 years of age, women and girls, and people living in the most socio‐economically disadvantaged than for applicants in the respective comparator groups. However, among people deemed eligible for NDIS support, differences by these socio‐demographic characteristics in the allocation (plan size) and spending (use of allocated funding) were less marked. The exception was that mean NDIS support spending was higher for women, particularly those with psychosocial disability, than would be expected on the basis of their mean plan size.

We found that socio‐demographic differences in eligibility rates for applicants with brain injury or stroke, intellectual disability, or autism were small, and larger for people with physical disability, psychosocial disability, or “other” disabilities, particularly for women and girls with physical or psychosocial disability. One reason for these differences could be the use of diagnostic lists to determine eligibility for some participants. These lists determine how much information is required for an application; for example, people with diagnostic conditions on list A^21^ (eg, level 2 autism diagnosis, which indicates need for substantial support) do not have to provide as much evidence as those on list B^22^ (eg, Parkinson disease) or those with health conditions included in neither list (eg, schizophrenia). The consequence is that eligibility assessments for people with some conditions are relatively rapid, while applicants without list A diagnoses must provide more information about how their condition affects their daily life.

The differences in eligibility for women and girls we found accord with the findings of other qualitative studies that identified barriers for women seeking individualised disability support.^8^ Although 50% of Australians aged 7–64 years with disabilities in 2018 were girls or women,^23^ only 43% of NDIS applicants and 41% of participants in 2022 were women or girls (Supporting Information, table 6). Some of the difference is explained by the larger number of boys with diagnoses of autism. It is increasingly recognised that gender‐related biases are barriers to being diagnosed with autism,^24^ and this could partly explain the NDIS differences we found.

We found small differences in plan size for women and girls and for people living in socio‐economically disadvantaged areas. Among women and girls, particularly those with psychosocial disability, we found that service and support spending was greater than expected from the allocated plan size. We found that the eligibility rate for women and girls with psychosocial disability was lower than for other applicants. Women with psychosocial disability deemed eligible may have greater support needs and use more NDIS services, consistent with findings that mean mental health scores are lower for women than men with psychological disability.^25^ Access to the NDIS should be further investigated to determine whether it leads to systematically different support needs by gender.

The eligibility differences by residential socio‐economic disadvantage we found could be attributed to the cost of gathering sufficient supporting medical evidence. This hypothesis would be consistent with findings that out‐of‐pocket costs are a barrier to medical specialist care in Australia.^26^ As regional and remote areas are more likely than metropolitan areas to be classified as socio‐economically disadvantaged, the differences could also reflect difficulties in service access outside metropolitan areas. Investigating the separate effects of remoteness and individual‐level socio‐economic position on access to and use of NDIS support should be investigated.

The consistently lower eligibility rates for people aged 55 years or older, particularly for people with physical disability (Supporting Information, table 6), are reflected in the younger age profile of NDIS participants than of all Australians with disability.^12^, ^23^ The reasons for these differences are unclear, but one could be the limited availability of disability support outside the NDIS.^4^ People may apply for NDIS support before they turn 65, as leaving their application until later risks age‐related ineligibility and receiving no disability support.

### Limitations

Our causal approach, including our use of causal mediation techniques,^27^, ^28^ allowed us to determine the proportion of the spending differences explained by differences in plan allocation size, providing a more nuanced assessment of inequalities across the budget allocation and spending process. However, despite having adjusted for key factors, residual confounding by factors for which data were unavailable is possible. For instance, we did not have information about applicants’ support needs beyond their prior source of disability support, primary disability group, and plan size. More information about functioning and support needs of NDIS applicants would be useful. Further, we could not examine all facets of inequality for people with disability; for example, barriers to NDIS access and use have been reported for people from culturally and linguistically diverse backgrounds and Indigenous people.^29^ Cultural and systemic barriers should be investigated. Finally, only the amount of personalised NDIS support spending was assessed; we could not determine whether the disability support and services used were appropriate for meeting individual needs.

## Conclusion

The NDIS is the most comprehensive national system of personalised, self‐directed disability support in the world. We report the first study based on unit record data for all people who have applied for and used NDIS support to quantify whether social inequalities are evident at different stages of the process. NDIS applicants in certain disability groups who are aged 55 years or older, women or girls, or living in socio‐economically disadvantaged areas are less likely to be deemed eligible for the NDIS than people in the corresponding comparator groups, but differences in plan size and use of personal NDIS budgets are less marked. One recommendation of the 2023 review of the NDIS was that eligibility should be determined by functional capacity rather than medical diagnosis.^30^ The review also recommended that gathering evidence for eligibility should be financed by the government rather than by applicants. This policy change could lead to applicants with a different socio‐demographic and disability profiles applying for NDIS support. Our analysis methods should be applied to evaluating the impact of policy changes on the social and economic differences we found. It is critical that changes to the NDIS do not perpetuate or exacerbate inequalities.

## Competing interests

All authors have conducted commissioned work for the Australian Department of Social Services (NDIS service use) and the Victorian Department of Families Fairness and Housing (inequalities in NDIS service use). George Disney, Yi Yang and Peter Summers have also undertaken work for the Queensland Department of Seniors, Disability Services, and Aboriginal and Torres Strait Islander Partnerships (NDIS service use in regional and remote Queensland).

## Data sharing

The data used in this study are available from the National Disability Insurance Agency upon request via a research agreement.

## Supporting information


Supplementary methods and results
