# Supplementary material for: Social inequalities in eligibility rates and use of the Australian National Disability Insurance Scheme, 2016–22: an administrative data analysis
Source: Med J Aust. 2025 Feb 16;222(3):135–43. doi: 10.5694/mja2.52594 (PMC11830222; doi:10.5694/mja2.52594)
Supplement: Supplementary file 1 — Supplementary methods and results [file MJA2-222-135-s001.pdf]

MJA
The Medical Journal of Australia

Supporting Information

Supplementary methods and results

This appendix was part of the submitted manuscript and has been peer reviewed.
It is posted as supplied by the authors.

Appendix to: Disney G, Yang Y, Summers P, et al. Social inequalities in eligibility rates and use of the Australian National Disability Insurance Scheme, 2016–22: an administrative data analysis. *Med J Aust* 2025; doi: 10.5694/mja2.52594.

Supplementary methods

1. Definitions

Table 1. Classes and categories of National Disability Insurance Scheme support[1]

Class	Category	Typical examples
Core	Assistance with daily life	Assistance with everyday needs, household cleaning or yard maintenance.
	Assistance with Social & Community Participation	A support worker to assist participation in social and community activities.
	Transport	Support that helps travelling to work or other places that will help achieve the goals in your plan.
	Consumables	Everyday items, such as continence products or low-cost assistive technology and equipment to improve independence and/or mobility.
Capacity building	Support coordination	This is a fixed amount for a support coordinator to help a participant use the plan.
	Improved life choices	Plan management support to help manage plan, funding and paying for services.
	Improved daily living	Assessment, training, or therapy to help increase skills, independence, and community participation.
	Increased social and community participation	Development and training to increase skills for participating in community, social and recreational activities.
	Finding and keeping a job	Employment-related support, training and assessments that help find and keep a job, such as the school leaver employment supports.
	Improved health and wellbeing	Exercise or diet advice to manage the impact of disability. The National Disability Insurance Scheme does not fund gym memberships.
	Improved living arrangements	Support to help find and maintain an appropriate place to live.
	Improved learning	Training, advice and help for moving from school to further education (e.g., university).
	Improved relationships	Support that helps develop positive behaviours and interact with others.
Capital	Home modifications	Home modifications such as installation of a handrail in a bathroom, or specialist disability accommodation for participants who require special housing because of their disability.
	Assistive technology	Equipment items for mobility, personal care, communication, and recreational inclusion such as wheelchairs or vehicle modifications.

Table 2. Variables and models used to estimate effects in the target trial emulation

Variables	Categories	Time of data collection	Model type when used as dependent variable*	Conditioned variables in model	Functional form (as independent variable)
Groups (exposure)					
Woman or girls (self-reported gender)	Indicator (yes; no)	Access request	Not predicted	-	2 categories
Live in socio-economically disadvantaged areas (areas in the lowest three IRSD deciles)	Indicator (yes; no)	Access request	Not predicted	-	pseudo-continuous (deciles)
Aged 55 years or older	Indicator (yes; no)	Access request	Not predicted	-	7 categories (see Table 3)
Access request					
Successful access request outcome	Indicator (yes; no)	End of access request process	Logistic regression	Confounder (previous source of disability support), exposure-confounder interaction	-
Budget allocation (plan size)					
Annualised plan size	Continuous (dollars)	Start of the eligible plan	Gamma regression (log link)	Confounders,† exposure-confounder interactions (with disability severity score and years in the NDIS before current plan)	Continuous
Use of services and supports					
Annualised spending	Continuous (dollars)	End of the eligible plan	Gamma regression (log link)	Confounders, exposure-confounder interactions (with disability severity score and years in the NDIS before current plan), and the predicted values of plan size	-
Confounders					
Indigenous (self-identified Aboriginal or Torres Strait Islander)	Indicator (yes; no)	Access request	Not predicted	-	2 categories
Living in regional and remote areas	Indicator (yes; no)	Access request	Not predicted	-	2 categories
Disability severity score	1 to 5; 6 to 10; 11 to 15	Scheme entry	Not predicted	-	3 categories
Years in NDIS before current plan	≤ 1 years; > 1 to 2 years; > 2 to 3 years; > 3 to 4 years; > 4 years	Start of the eligible plan	Not predicted	-	6 categories
Had any of the disability support related experience listed in Table 3	Indicator (yes; no)	Scheme entry	Not predicted	-	2 categories
Previous source of disability support	Australian government; state government; none	Scheme entry	Not predicted	-	3 categories

IRSD = Index of Relative Socio-economic Disadvantage.[2]

* Pre-exposure variables were not predicted. † In the analysis of plan size and spending, exposures other than the exposure of interest were included as confounders. We did not include remoteness when area-based socio-economic status was the exposure of interest because both characteristics are defined by postcode of residence and are therefore related; adjustment for remoteness could mask part of the effect of area-based socio-economic disadvantage.

2. Emulation of target trials using NDIS data

We employed the counterfactual outcomes approach to causal inference, in which the likelihood of a particular outcome is estimated for an individual in alternative scenarios. For instance, we consider the likelihood of an individual experiencing a certain outcome or having a certain plan size if they were in the comparator group instead of the group of interest. These alternative scenarios and their corresponding outcomes are referred to as counterfactual outcomes (i.e., contrary to fact).[3] By adopting this approach, we seek to emulate a randomised controlled trial, which is widely recognized as the ideal study design for generating data for causal inferences.

To approximate randomisation as closely as possible, we adjusted for factors that co-occur with the exposure and influence the outcome (Boxes 3 and 4, step 1).

Estimating effects using Monte Carlo simulation-based g-computation

Assuming conditional exchangeability (i.e., no unmeasured confounding for the exposure-outcome, mediator-outcome, and exposure-mediator relationships), we estimated the total causal effect of being in a group of interest on access request success rate ($TCE_{\text{access rate}}$, Box 3):

1. Fit outcome regression model to the observed data (see Table 3 for model details).
2. Use fitted model to predict the outcomes (whether access request is successful or not) for all individuals under the following scenarios corresponding to the two arms specified in the target trial, given the values of their confounder vector *C*.
    a. Predict access request outcome, given all individuals are in the comparator group. Take the mean of the outcomes (Y_0).
    b. Predict access request outcome, given all individuals are in the exposure group. Take the mean of the outcomes (Y_1).
3. Repeat steps 1 and 2 ten times and calculate the mean outcome prediction across the whole sample.
4. Calculate the difference between the two means:

$$TCE_{\text{access rate}} = Y_1 – Y_0$$

To estimate the total causal effect of being in an inequality group on plan size and spending ($TCE_{\text{plan size}}$ and TCE_{spending}, Box 4), and to decompose the spending inequality (TCE_{spending}) into spending differences due to plan size inequality (IIE), and remaining differences that were not explained by different plan sizes (IDE), we followed the steps below:

1. Fit mediator and outcome regression models to the data. Details of the variables and models fitted are reported in Table 3.
2. For each individual, draw plan size values from the fitted mediator distributions corresponding to the exposure value (i.e., inequality group or comparator) of a given target trial arm, given the values of their confounder vector *C*:
    a. Draw plan size values, given all individuals are in the comparator group. Take the mean of the plan sizes (M_0).
    b. Draw plan size values, given all individuals are in the inequality group. Take the mean of the plan sizes (M_1).
3. Using the fitted outcome model, for each individual, predict the outcome given the values of their covariate vector *C*, the relevant exposure value and the mediator draw.
    a. Predict spending values, given all individuals are in the comparator group and have the plan size distribution of the comparator group. Take the mean spending (Y_{0M0}).
    b. Predict spending values, given all individuals are in the exposure group and have the plan size distribution of the inequality group. Take the mean spending (Y_{1M1}).
    c. Predict spending values, given all individuals are in the exposure group, with the plan size distribution of the comparator group. Take the mean spending (Y_{1M0}).

4. Repeat steps 1 to 3 ten times and calculate the mean outcome predictions.
5. Calculate causal contracts we are interested in:
    a. TCE $_{\text{plan size}}$ = $M_1 – M_0$
    b. TCE $_{\text{spending}}$ = $Y_{1M1} – Y_{0M0}$
    c. IIE = $Y_{1M1} – Y_{1M0}$
    d. IDE = $Y_{1M0} – Y_{0M0}$

We repeated the above steps for each exposure group. Standard errors and 95% confidence intervals were obtained using non-parametric bootstrapping (500 bootstrap samples). Example analysis code is available at https://github.com/YiYang368/NDIS_Inequalities.

Gender categories

The gender category of “not women and girls” included people whose recorded gender was not women or girls, predominantly boys and men (97.5% of the applicants and 98.1% of participants included in our analysis who were not women or girls). It also included people with “unknown” gender, including those who reported their gender as “other” or preferred not to disclose their gender. Because of the small proportion of people in the “unknown” gender category and the lack of further information about this group, we decided to group them with boys and men.

Supplementary results

Figure 1. Flowchart of applicants and participants

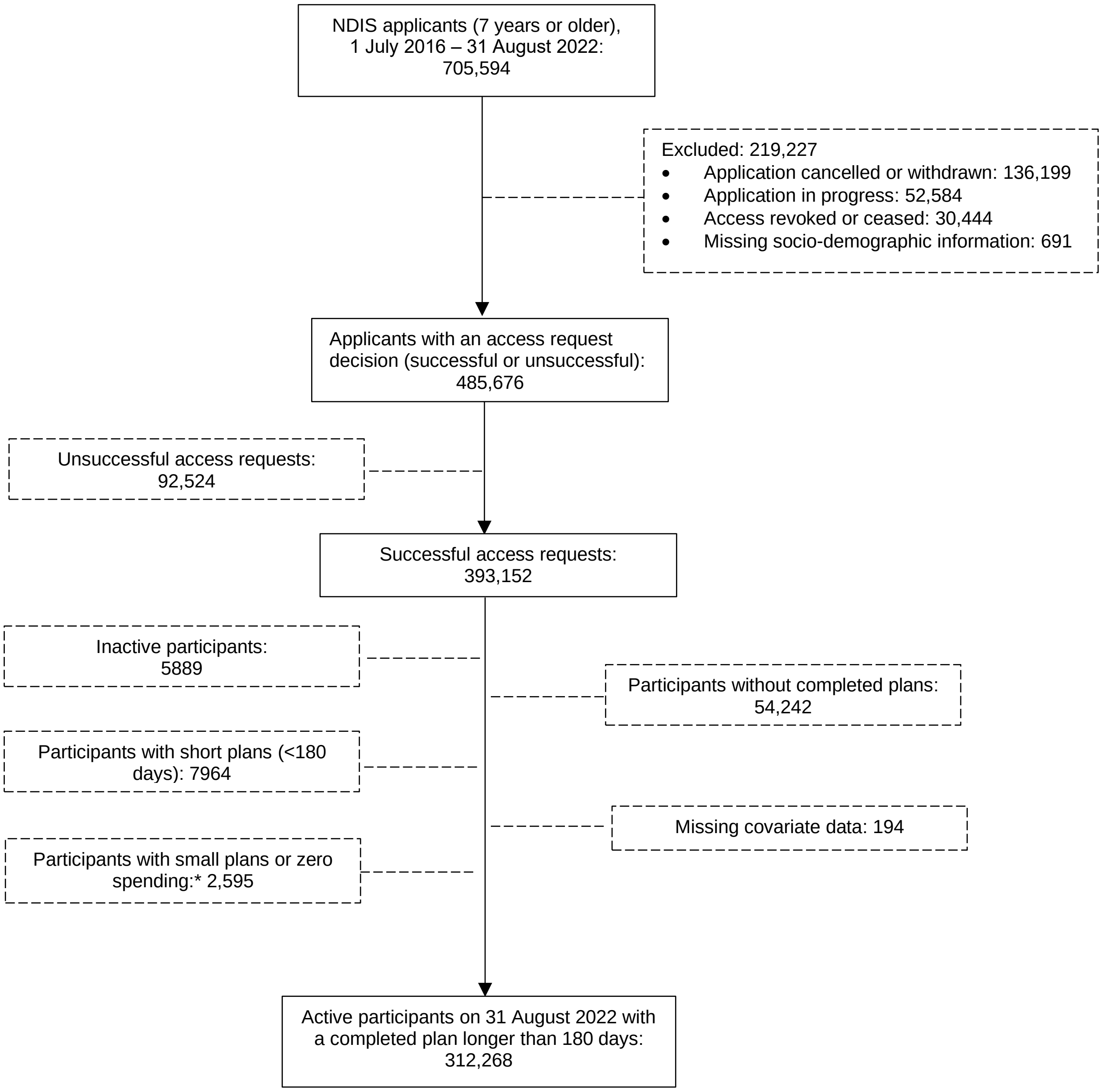

* Annualised budget less than $1000 for all support classes.

Table 3. Differences in access request success rates between the exposure groups and the corresponding comparator groups

	Differences in successful outcomes per 1000 access requests (95% CI)*		
**Disability group**	**Aged 55 years or older**	**Women or girls**	**Living in socio-economically disadvantaged areas**
**All disability types**	**–44 (–47 to –42)**	**–35 (–37 to –33)**	**–32 (–34 to –30)**
Sensory	–35 (–45 to –25)	24 (16 to 32)	–34 (–43 to –25)
Autism	–90 (–114 to –67)	9 (7 to 11)	–2 (–5 to 0)
Intellectual other than autism	–30 (–37 to –24)	–4 (–7 to –1)	–5 (–8 to –3)
Physical	–235 (–242 to –227)	–145 (–152 to –138)	–86 (–93 to –78)
Psychosocial	–40 (–47 to –32)	–83 (–89 to –77)	–39 (–45 to –33)
Brain injury or stroke	–40 (–47 to –32)	–21 (–29 to –14)	–15 (–23 to –7)
Other	69 (61 to 77)	–22 (–30 to –14)	–67 (–76 to –58)

CI =confidence interval.
* Adjusted for prior source of disability support.

Table 4. National Disability Insurance Scheme (NDIS) participant characteristics by primary disability group (312,268 active participants at 31 August 2022)

	Disability group						
**Characteristic**	**Sensory**	**Autism**	**Intellectual other than autism**	**Physical**	**Psycho-social**	**Brain injury or stroke**	**Other**
Number of participants	20560 [6.6%]	88609 [28.4%]	76248 [24.4%]	32935 [10.5%]	45056 [14.4%]	20476 [6.6%]	28384 [9.1%]
Age group, plan start (years)							
7–14	3338 (16.2%)	47150 (53.2%)	10414 (13.7%)	2905 (8.8%)	202 (0.4%)	416 (2.0%)	1311 (4.6%)
15–18	1480 (7.2%)	17096 (19.3%)	8618 (11.3%)	1805 (5.5%)	352 (0.8%)	352 (1.7%)	804 (2.8%)
19–24	1522 (7.4%)	12819 (14.5%)	12919 (16.9%)	2410 (7.3%)	1932 (4.3%)	668 (3.3%)	1004 (3.5%)
25–34	2354 (11.4%)	6949 (7.8%)	14278 (18.7%)	3929 (11.9%)	6706 (14.9%)	1582 (7.7%)	1807 (6.4%)
35–44	2430 (11.8%)	2588 (2.9%)	10306 (13.5%)	4579 (13.9%)	10464 (23.2%)	2736 (13.4%)	3304 (11.6%)
45–54	3687 (17.9%)	1324 (1.5%)	9811 (12.9%)	6275 (19.1%)	12663 (28.1%)	5123 (25.0%)	5906 (20.8%)
55–64	5749 (28.0%)	683 (0.8%)	9902 (13.0%)	11032 (33.5%)	12737 (28.3%)	9599 (46.9%)	14248 (50.2%)
Gender (women and girls)	10499 (51.1%)	23800 (26.9%)	33125 (43.4%)	14751 (44.8%)	21977 (48.8%)	7563 (36.9%)	15410 (54.3%)
Living in socio-economically disadvantaged areas*	909 (4.4%)	5139 (5.8%)	6042 (7.9%)	1778 (5.4%)	3320 (7.4%)	1623 (7.9%)	1052 (3.7%)
Living in regional or remote areas	5887 (28.6%)	24158 (27.3%)	25534 (33.5%)	9898 (30.1%)	13465 (29.9%)	6631 (32.4%)	7768 (27.4%)
Previous source of support	5937 (28.9%)	28551 (32.2%)	26843 (35.2%)	10999 (33.4%)	12647 (28.1%)	7146 (34.9%)	9163 (32.3%)
Australian government	2295 (11.2%)	7591 (8.6%)	5423 (7.1%)	992 (3.0%)	8773 (19.5%)	1523 (7.4%)	1885 (6.6%)
State government	5746 (27.9%)	32622 (36.8%)	52071 (68.3%)	17823 (54.1%)	15048 (33.4%)	9092 (44.4%)	11043 (38.9%)
No prior support	12519 (60.9%)	48396 (54.6%)	18754 (24.6%)	14120 (42.9%)	21235 (47.1%)	9861 (48.2%)	15456 (54.5%)
Disability severity score							
1–5	15471 (75.2%)	11517 (13.0%)	9387 (12.3%)	5087 (15.4%)	2001 (4.4%)	1533 (7.5%)	1651 (5.8%)
6–10	4540 (22.1%)	56651 (63.9%)	36709 (48.1%)	12688 (38.5%)	28211 (62.6%)	9393 (45.9%)	11844 (41.7%)
11–15	549 (2.7%)	20441 (23.1%)	30152 (39.5%)	15160 (46.0%)	14844 (32.9%)	9550 (46.6%)	14889 (52.5%)
Time in NDIS prior to current plan (years)							
One or less	8713 (42.4%)	32938 (37.2%)	16814 (22.1%)	8956 (27.2%)	20402 (45.3%)	7009 (34.2%)	9505 (33.5%)
More than 1 to 2	5085 (24.7%)	20207 (22.8%)	17396 (22.8%)	7961 (24.2%)	10980 (24.4%)	4845 (23.7%)	6416 (22.6%)
More than 2 to 3	3673 (17.9%)	16853 (19.0%)	18796 (24.7%)	7427 (22.6%)	7719 (17.1%)	4375 (21.4%)	5986 (21.1%)
More than 3 to 4	2318 (11.3%)	13108 (14.8%)	15575 (20.4%)	5632 (17.1%)	4390 (9.7%)	2917 (14.2%)	4313 (15.2%)
More than 4	771 (3.8%)	5503 (6.2%)	7667 (10.1%)	2959 (9.0%)	1565 (3.5%)	1330 (6.5%)	2164 (7.6%)

	Disability group						
**Characteristic**	**Sensory**	**Autism**	**Intellectual other than autism**	**Physical**	**Psycho-social**	**Brain injury or stroke**	**Other**
Disability accommodation programs							
Ever been a Younger People in Residential Aged Care Strategy participant	30 (0.1%)	29 (<0.1%)	681 (0.9%)	425 (1.3%)	687 (1.5%)	1657 (8.1%)	1822 (6.4%)
Ever received funds for supported independent living	132 (0.6%)	3661 (4.1%)	15421 (20.2%)	3218 (9.8%)	3527 (7.8%)	2994 (14.6%)	2140 (7.5%)
Ever received funds for specialist disability accommodation	102 (0.5%)	2509 (2.8%)	11633 (15.3%)	3354 (10.2%)	1347 (3.0%)	2611 (12.8%)	2281 (8.0%)
Received a trial plan prior to 30 June 2016	733 (3.6%)	5785 (6.5%)	5586 (7.3%)	1684 (5.1%)	1779 (3.9%)	751 (3.7%)	1164 (4.1%)

Table 5. Estimated plan size and spending differences for 312,268 active National Disability Insurance Scheme (NDIS) participants (31 August 2022), by primary disability group

Disability group	Effects	Plan size/spending differences to $ thousands (95% CI) [a]	
		**Women and girls**	**Live in socio–economically disadvantaged areas**
All disability types	TCE_{plan}	–2.4 (–3.2 to –1.7)	–0.8 (–1.6 to 0.0)
	$TCE_{spending}$	–0.6 (–1.3 to 0.2)	–0.8 (–1.5 to 0.0)
	IIE	–2.2 (–2.9 to –1.6)	–0.9 (–1.6 to –0.2)
	IDE	1.7 (1.4 to 2.0)	0.1 (–0.2 to 0.5)
Sensory	TCE_{plan}	–0.0 (–0.8 to 0.7)	2.4 (1.5 to 3.3)
	$TCE_{spending}$	0.3 (–0.4 to 1.1)	2.0 (1.2 to 2.9)
	IIE	–0.1 (–0.7 to 0.5)	1.6 (0.9 to 2.4)
	IDE	0.4 (0.0 to 0.8)	0.4 (–0.0 to 0.8)
Autism	TCE_{plan}	0.9 (–0.3 to 2.0)	–0.8 (–1.8 to 0.2)
	$TCE_{spending}$	1.1 (0.1 to 2.1)	–0.3 (–1.2 to 0.6)
	IIE	0.7 (–0.2 to 1.7)	–0.6 (–1.5 to 0.2)
	IDE	0.4 (–0.0 to 0.8)	0.4 (–0.0 to 0.8)
Intellectual other than autism	TCE_{plan}	–2.9 (–4.5 to –1.3)	–3.0 (–4.7 to –1.2)
	$TCE_{spending}$	–1.5 (–3.0 to 0.0)	–3.1 (–4.9 to –1.4)
	IIE	–2.7 (–4.1 to –1.3)	–2.4 (–4.0 to –0.9)
	IDE	1.2 (0.5 to 1.9)	–0.7 (–1.5 to –0.0)
Physical	TCE_{plan}	–8.8 (–11.7 to –6.0)	–4.4 (–7.3 to –1.5)
	$TCE_{spending}$	–8.3 (–11.2 to –5.5)	–4.5 (–7.4 to –1.6)
	IIE	–8.3 (–10.9 to –5.7)	–4.2 (–6.9 to –1.6)
	IDE	–0.0 (–1.3 to 1.3)	–0.3 (–1.5 to 1.0)
Psychosocial	TCE_{plan}	1.0 (–0.2 to 2.3)	4.4 (2.9 to 5.8)
	$TCE_{spending}$	2.9 (1.7 to 4.0)	3.6 (2.3 to 4.9)
	IIE	0.5 (–0.5 to 1.6)	3.5 (2.4 to 4.7)
	IDE	2.3 (1.8 to 2.9)	0.1 (–0.5 to 0.7)
Brain injury or stroke	TCE_{plan}	6.8 (3.2 to 10.4)	–7.2 (–11.1 to –3.3)
	$TCE_{spending}$	6.7 (3.2 to 10.3)	–6.0 (–9.9 to –2.1)
	IIE	5.5 (2.3 to 8.7)	–6.2 (–9.7 to –2.8)
	IDE	1.2 (–0.2 to 2.7)	0.2 (–1.5 to 1.9)
Other	TCE_{plan}	–3.0 (–5.8 to –0.1)	3.9 (1.0 to 6.7)
	$TCE_{spending}$	–0.7 (–3.4 to 2.0)	3.6 (0.8 to 6.4)
	IIE	–2.4 (–4.7 to 0.0)	2.9 (0.5 to 5.3)
	IDE	1.6 (0.5 to 2.8)	0.7 (–0.5 to 1.9)

CI, confidence interval; TCE, total causal effect; IIE, interventional indirect effect; IDE, interventional direct effect.

[a] Estimates were adjusted for age, remoteness of living, severity of disability, years in the NDIS, participation in disability accommodation programs or received NDIS trial plans, and source of disability support before the NDIS. When one of the inequality groups was the exposure of interest, other inequality exposures were included as confounders. We did not adjust for remoteness of living when area-based socioeconomic status was the inequality of interest, because both variables were defined using area of residence and were closely related, adjustment of remoteness may mask some effect of area-based socioeconomic disadvantage on plan size and spending.

Table 6. Age and sex profiles of National Disability Insurance Scheme (NDIS) applicants and Australians with disability aged 7–64 years (estimated from Survey of Disability, Ageing and Carers 2018[4])

Characteristic	Australians with disability, 2018[a]	Australians with severe or profound disability, 2018[b]	Total NDIS applicants with an outcome by 31 August 2022 [c]	Successful NDIS applicants by 31 August 2022[c]
Estimated number of people	2288290	636670	485676	393152
Age (years)				
7 to 14	231559 (10.1%)	125815 (19.8%)	126268 (26.0%)	111897 (28.5%)
15 to 18	152707 (6.7%)	61112 (9.6%)	41647 (8.6%)	37611 (9.6%)
19 to 24	134381 (5.9%)	42707 (6.7%)	36463 (7.5%)	32432 (8.2%)
25 to 34	259725 (11.4%)	65608 (10.3%)	50967 (10.5%)	43616 (11.1%)
35 to 44	320087 (14.0%)	64085 (10.1%)	56232 (11.6%)	45570 (11.6%)
45 to 54	493832 (21.6%)	110588 (17.4%)	73712 (15.2%)	55973 (14.2%)
55 to 64	696001 (30.4%)	166755 (26.2%)	100387 (20.7%)	66053 (16.8%)
Gender (female)	1143245 (50.0%)	303737 (47.7%)	210274 (43.3%)	160198 (40.7%)

NDIS, National Disability Insurance Scheme; SDAC, Survey of Disability, Ageing and Carers.

[a] SDAC definition of disability: at least one limitation, restriction or impairment to everyday activities, for at least 6 months.

[b] SDAC definition of severe or profound disability: always or sometimes need help with 1 or more of 3 core activities (self-care, mobility, and communication).

[c] NDIS definition of disability: permanent loss or reduction in functional capacity to undertake 1 or more of 6 core activities.

References

1. National Disability Insurance Agency. Support budgets in your plan: core supports budget. Updated 24 Apr 2024. https://www.ndis.gov.au/participants/using-your-plan/managing-your-plan/support-budgets-your-plan#core-supports-budget (viewed Jan 2025).
2. Australian Bureau of Statistics. IRSD. In: 2033.0.55.001. Census of Population and Housing: Socio-Economic Indexes for Areas (SEIFA), Australia, 2016. 27 Mar 2018. https://www.abs.gov.au/ausstats/abs@.nsf/Lookup/by%20Subject/2033.0.55.001~2016~Main%20Features~IRSD~19 (viewed Jan 2025).
3. Hernán MA, Robins JM. Causal inference: what if. Boca Raton: Chapman & Hall/CRC, 2020.
4. Australian Bureau of Statistics . Disability, ageing and carers, Australia: summary of findings, 2018. 24 Oct 2019. https://www.abs.gov.au/statistics/health/disability/disability-ageing-and-carers-australia-summary-findings/2018 (viewed Nov 2024).
